# Eco-Friendly and Cost-Effective High-Density Polyethylene-Based Composites: Optimizing Wood–Plastic Composites for Enhanced Performance

**DOI:** 10.1021/acsomega.4c06422

**Published:** 2025-02-14

**Authors:** Ricardo S. Ferreira, Guilherme A. M. Jesus, Johny P. Monteiro, Alessandro F. Martins, Rodolfo K. Tessari, Elton G. Bonafé

**Affiliations:** † Laboratory of Materials, Macromolecules, and Composites (LaMMAC), Federal University of Technology − Paraná (UTFPR), Apucarana, PR 86812-460, Brazil; ‡ Department of Civil Engineering, Federal University of Technology − Paraná (UTFPR), Apucarana, PR 86812-460, Brazil; § Department of Chemistry and National Institute for Materials Advancement, Pittsburgh State University (PSU), Pittsburgh, Kansas 66762-7500, United States

## Abstract

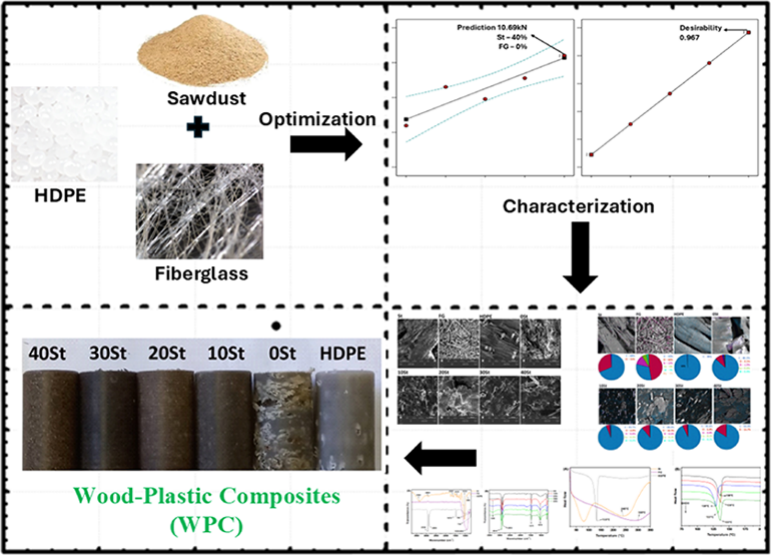

Petroleum-based products have been linked to global warming. In this context, wood-plastic composites (WPCs) emerge as an economically and ecologically attractive alternative. Therefore, for the first time, this study aims to produce, optimize, and characterize high-density polyethylene (HDPE)-based WPC loaded with sawdust (St) and fiberglass (FG) without compatibilizers. The amounts of St (0–40%, w/w) and FG (0–40% w/w) were optimized for compressive strength using a Simplex Lattice mixture design. The WPCs were extensively characterized. The composites demonstrated densities ranging from 780 to 987 kg/m^3^, low moisture retention (0.83–2.45%), and mechanical properties of 0.97–10.89 kN. Scanning electron microscopy (SEM) micrographs showed homogeneous materials in mixtures containing St. The random distribution of Si on the material surfaces was identified by energy dispersive spectroscopy (EDS). Furthermore, the optimization indicated that the WPC loaded with 40% St (40 St) leads to the most compression-resistant (11.034 kN) composite. The results suggest a 17.3% greater strength than that of the control (8.93 kN). On the other hand, a simple calculation indicates a 37% reduction in the production cost for the optimized 40 St (US$ 0.53/kg) amount compared to pure HDPE (US$ 0.84/kg). Additionally, substituting HDPE with St (amount of 40 St) could reduce equivalent carbon emissions. Thus, the results suggest that 40 St WPC has potential market applications. The new technology could contribute to environmental sustainability, lowering production costs.

## 1 Introduction

A representative panel of the United Nations on climate change indicates that the global warming observed in recent years results from CO_2_ emissions.^1^ Although plastic materials play a crucial role in developing new products, the process used to create synthetic plastics increases the level of CO_2_ emissions. Therefore, composites formed from synthetic and natural polymers have emerged as a promising alternative. Wood-plastic composites (WPCs) are materials made from a combination of a polymer matrix and wood-based reinforcement.^2^ The polymer matrix is generally composed of thermoplastic such as low-density polyethylene (LDPE), high-density polyethylene (HDPE), polypropylene (PP), and poly(vinyl chloride) (PVC).^3^ This study focuses on HDPE, which exhibits resistance to various solvents, a high strength-to-density ratio, and high specific strength.^4,5^ WPCs are often used as outdoor decking, railings, park benches, automotive door handles and headrests, fences, door and window frames, wood panel structures, and indoor furniture.^6,7^

Wood stores carbon through the capture of CO_2_ from the environment, which contributes to reducing global warming. This renewable material is widely used across various market segments.^8^ Recent data indicate global wood consumption could reach 2.2 billion cubic meters by 2030.^9^ In Brazil, eucalyptus cultivation is prominent, with 5.5 million ha planted and an estimated productivity of 39 m^3^/ha/year (Embrapa). However, each log processed into boards generates an average of 6% (w/w) residual sawdust. The St, composed of cellulose, hemicellulose, and lignin,^10^ exhibits desirable mechanical properties, low density, and high plasticity.^11,12^ In addition to natural fibers, synthetic reinforcements can enhance WPCs’ properties. Fiberglass (FG) comprises silica particles distributed in an amorphous silicon–oxygen network with filament diameters ranging from 5 to 24 μm. FG offers a low coefficient of thermal expansion, tensile strength, and vibration resistance, retention of mechanical properties at high temperatures, high elongation at break, ease of processing, and low cost.^13^ Thus, producing WPC reinforced with natural and synthetic materials makes the strategy economically and environmentally viable.

WPCs were produced through a combination of various polymers and reinforcing materials. Zhao et al. produced WPCs from a blend of poly(lactic acid) and natural pine fibers (30% w/w) modified with epoxy (0.5–4% w/w).^10^ A composite based on recycled HDPE (45–78.75% w/w) enriched with pine sawdust residue (11.25–45% w/w) formed WPCs compatibilized with Dupont Fusbond E365 (10% w/w).^14^ Natural fibers (RedWood, Scotch pine) (40–60% w/w) combined with HDPE (37.5–57.5% w/w) produced WPC linked to maleic anhydride (2% w/w).^5^ Pratheep et al. created environmentally friendly WPCs using corn cob powder (0–25% w/w) and coconut fiber powder (0–25% w/w) mixed with PVC and stabilized with maleic polyethylene.^15^ All of the WPCs contain compatibilizers. However, to the best of our knowledge, no HDPE/St/FG-based WPCs have been reported without compatibilizers.

Thus, for the first time, this study aimed to produce, optimize, and characterize HDPE-based WPC enriched with different proportions of St and FG without using compatibilizer agents. The St and FG contents were optimized for compressive strength utilizing a Simplex Lattice mixture design. The composites were extensively characterized through density, water absorption, mechanical properties, SEM, differential scanning calorimetry (DSC), and infrared spectroscopy (FTIR). Additionally, principal component analysis (PCA) was used to evaluate the similarity between the measurements.

## 2 Materials and Methods

### 2.1 Materials

The high-density polyethylene (HDPE), fiberglass (FG), and eucalyptus sawdust (St) commercial precursors were obtained from Cimflex Industria & Comércio de Plástico LTDA, Casa do Forro e Decor (Maringá-PR), and Serraria Rebenic Madeiras from Jandaia do Sul-PR, respectively.

### 2.2 Preparation of the WPCs

The WPCs were obtained from a mixture of HDPE and reinforcement precursors (FG and St). The levels of HDPE were kept constant at 60% w/w. The weight St/FG ratios were optimized for compressive strength response using a Simplex Lattice mixture design. The total content of St and FG is 40 g (100%). Table 1 displays the St and FG amounts ranging from 0–100% w/w (0–40 g) utilized to yield the composites. Thus, WPCs were named 0, 10, 20, 30, and 40 St. 0 St is the sample composed of 100% fiberglass, while 40 St is the sample comprising 100% St. The St was dried at 40 °C for 48 h (to remove moisture), ground, and homogenized using 60 mesh sieves. The FG was ground in an iron mill and sieved to 60 mesh. The commercial HDPE granules and different St/FG proportions were extruded at 200 °C to produce the WPCs.

**Table 1:** Composition and Simplex Lattice Design of the WPCs^a^

WPC	HDPE (g/% w/w)	St (g/% w/w)	FG (g/% w/w)	Compressive strength
0 St	60/60	–	40/40	0.97^d^ ± 0.04
10 St	60/60	10/10	30/30	5.7^c^ ± 0.60
20 St	60/60	20/20	20/20	5.63^c^ ± 0.67
30 St	60/60	30/30	10/10	7.90^b^ ± 0,21
40 St	60/60	40/40	–	10.89^a^ ± 0.36
HDPE^b^	100/100	–	–	8.93^b^ ± 0.47

a The weight content (%, w/w) of St/FG mixture was expressed in % w/w in 100 g. St: sawdust; FG: Fiberglass; (HDPE: high-density polyethylene. The WPC was developed in triplicate.

b Not applied in the experimental design.

### 2.3 Density

The density of the WPCs was calculated using eq 1, assuming a density of water of 1000 kg/m^3^.
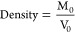
1

Where M_0_ is the mass (kg) of the material dried at 40 °C for 48 h in an oven, and V_0_ is the sample volume (m^3^).

### 2.4 Water Absorption

Water absorption of the materials was determined after immersion for 24 h in distilled water. The dry materials (40 °C for 48 h) were weighed (M_0_) and transferred to polyethylene containers possessing 50 mL of distilled water. After 24 h, the materials were removed from the recipient, dried with a paper towel, and weighed. The amount of absorbed mass was calculated using eq 2
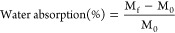
2where M_f_ and M_0_ are related to the final (after 24 h) and initial (previously dried) masses (g).

### 2.5 Mechanical Properties

Mechanical property tests were conducted using a CMT6104 electronic universal testing machine (MTS Industrial Systems Co., Ltd., Shanghai, China). An impact testing machine (XJJ-5, Jinjian Testing Instrument Co., Ltd., Chengde, China) was employed to perform the compression test by GB/T 1043.1–2008. The sample dimensions were 100 mm × 10 mm × 7 mm, and the tests were carried out in five replicates.

### 2.6 Scanning Electron Microscopy (SEM)

The surface morphology of the composites was investigated by using scanning electron microscopy (SEM). The materials were sputter-coated (10 nm) with a gold–palladium alloy (Polaron SC 7620 Sputter Coater, Quorum Technologies, Newhaven, UK) at 10–15 mA under a vacuum of 130 mTorr. The SEM (JSM-6500F, field emission scanning electron microscope, JEOL, Japan) was operated at an accelerating voltage of 5 kV.

### 2.7 Differential Scanning Calorimetry (DSC)

DSC curves were obtained by using a Shimadzu DSC60 Plus instrument (Japan) with a heating rate of 10 °C/min, from 20 to 350 °C, under an argon purge of 50 mL/min.

### 2.8 Fourier Transformed Infrared Spectroscopy (FTIR)

FTIR spectra for the WPCs were obtained using an Agilent infrared spectrophotometer (Cary 30, USA). KBr pellets (100 mg) containing approximately 3 mg of the material were analyzed between 4000 and 400 cm^–1^, with a resolution of 4 cm^–1^ and 64 scans.

### 2.9 Statistical Analysis

The data were analyzed using PAST software, with variance analysis (ANOVA) and Tukey’s test at a 5% significance level. Optimization studies were conducted by using Design Expert 7.0 software. In addition, PCA evaluated the differences and similarities within the data obtained from various measurements. The results from the different analyses were standardized to 0 and 1, thereby avoiding biases related to individual magnitudes

## 3 Results and Discussion

### 3.1 Preparation of the WPC

**Figure 1:**
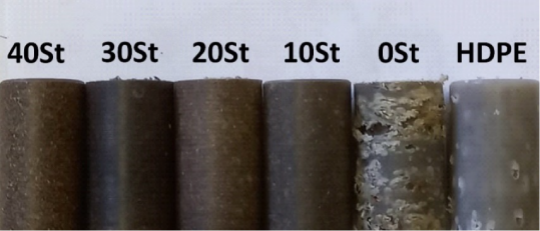
Digital images of the composites HDPE.

Figure 1 presents a digital image of extruded and machined WPCs in a cylindrical shape. The image displays all formulations (40 St, 30 St, 20 St, 10 St, 0 St) and the control (HDPE 100% w/w) without compatibilizers.

Homogeneous materials are obtained by increasing the St content. Conversely, the 0 St and HDPE composites exhibit porous surfaces with a low homogeneity. Some imperfections on the lateral WPC surfaces can be attributed to the production method. The extrusion process involves a linear continuous screw under controlled heating. HDPE-based polymeric materials become viscous at softening temperature (80–130 °C). The high viscosity prevents the escape of air bubbles, resulting in free volume within the material and heterogeneity (Figure 1). The highest incidence of free volume occurred in the 0 St material (Figure 1), which is composed of FG (40% w/w) and HDPE (60% w/w).

The material composition supports a low compatibility between the precursors and composite heterogeneity. FG presents silanol groups (Si–OH) on its surface, which confers hydrophilicity. FG primarily interacts through hydrogen bonds. In contrast, the HDPE, composed of ethylene repeating units, is hydrophobic and mainly interacts through London dispersion.^16^ Lu and coworkers report the weak interfacial adhesion between hydrophilic and hydrophobic matrices, resulting in low-strength composites.^17^

However, adding St to the mixture produced homogeneous composites without defects. St mainly comprises cellulose, hemicellulose, and lignin.^18^ Cellulose is a predominantly linear polysaccharide with carbon, hydrogen, and oxygen atoms.^19^ The hydroxyl groups in the polysaccharide interact through hydrogen bonding with FG, acting as a compatibilizer in the composite. Furthermore, the linear arrangement of cellulose chains allows macromolecular entanglements in the composites, resulting in compact and stiff materials.

### 3.2 Optimization of the Composites

The WPCs were produced from a matrix (HDPE) and reinforcement materials (St and FG) without compatibilizers. Experimental studies suggest a WPC composition of 60% (w/w) HDPE and 40% (w/w) reinforcement material. The St and FG contents (0–40% w/w) were optimized for compressive strength using Simplex Lattice mixture design. The compressive strength data fit the linear order model. The analysis of variance (ANOVA) exhibited in Table 2 shows high F values and p-values <0.05. These values indicate the significance of the linear model and linear component of the mixture. The model’s determination coefficients (R-squared, predicted R-squared, adjusted R-squared, and adequate precision) also suggest the model’s significance. The R-squared value close to 0.9 proposes a strong correlation between the predicted and experimental outcomes; the predicted R-squared of 0.7143 is consistent with the adjusted R-squared of 0.8370; and a signal-to-noise ratio of 9.801 implies that the model can make effective predictions within the evaluated space.^20^

**Table 2:** ANOVA of the Simplex Lattice Mixture Design for Composite Optimization^a^

	Tensile-Compression			
Source	Sum of squares	Mean square	F-values	p-values
Linear Model	64.86	64.86	26.68	0.0067
Linear Mixture	64.86	24.86	26.68	0.0067
Residual	9.72	9.72		
Cor Total	74.58			
R-Squared	0.8696			
Adj R-Squared	0.8370			
Pred R-Squared	0.7143			
Adeq precision	9.801			

a Predictted R-Squared: Adj R-Squared; Adjustted R-squared: Adj R-Squared; Adequated precision: Adeq precision.

Equation 3 provides possible predictions from the model. Both variables of the model (St and FG) exhibit synergy with the optimized response. However, it is notable that the influence of the St component is predominant. The St contribution is associated with a coefficient 5.7 times greater than that of the FG (1.8755). Thus, increasing the amount of St in the composite improves the compressive strength responses.
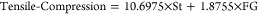
3

**Figure 2:**
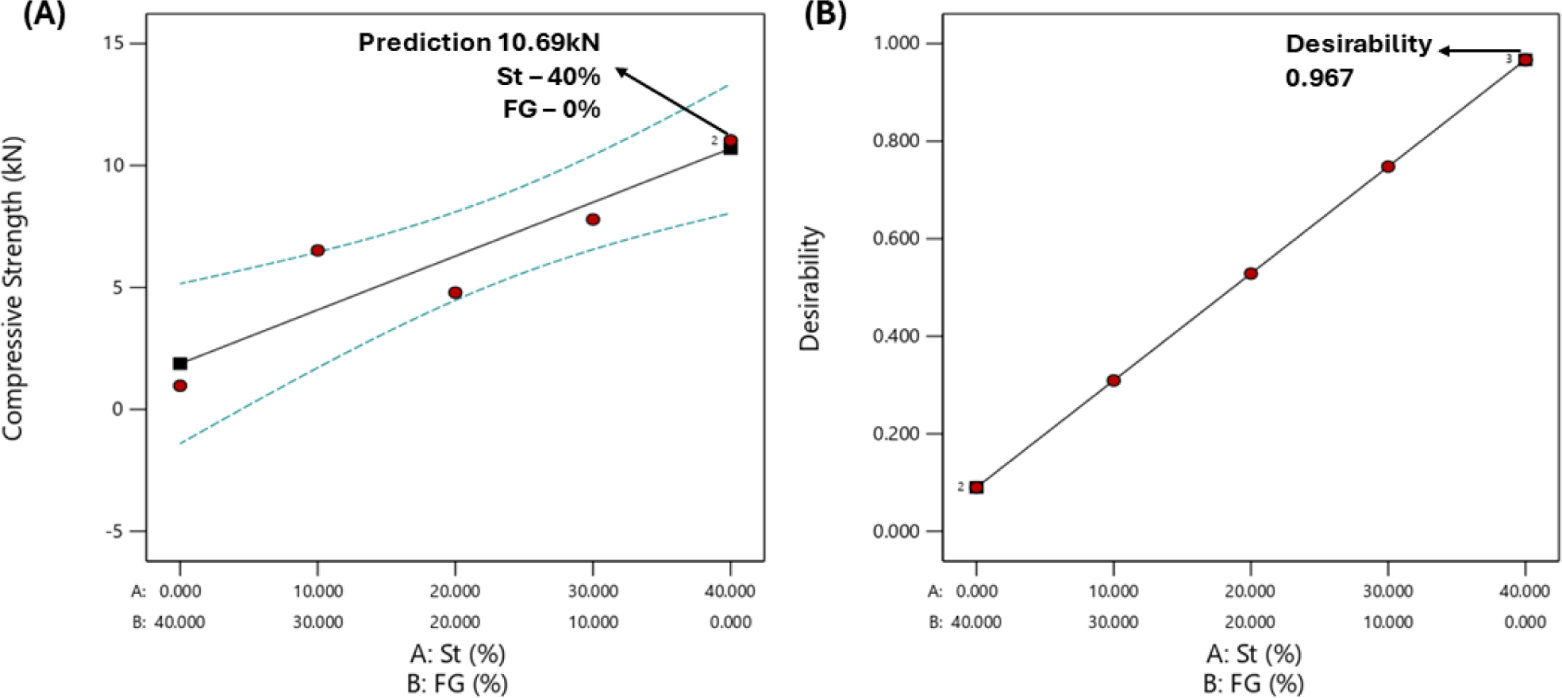
Point prediction (A) and desirability (B) of the optimization process.

Furthermore, the optimization process was evaluated by using the desirability function. The function was applied based on the concentration range of each component in the matrix, St 0–40% (w/w) and FG 0–40% (w/w), aiming to maximize the response (compressive strength). Figure 2A illustrates the response behavior with the concentration alteration of St and FG. An increase in the St concentration contributes to obtaining more resistant WPC. A higher content of St in the mixture than FG results in materials with greater compressive strength. In addition, the function indicates a theoretical compressive strength of 10.69 MPa for a mixture of 40% w/w St and FG 0% w/w with a desirability of 96.7% (Figure 2B). These results are consistent with the experimental measurements, which was 10.89 MPa.

Therefore, the optimization suggests that the St40 composite is the most resistant. These results highlight an important economic aspect. Replacing 40% of HDPE with natural St fibers would reduce the final production costs. A brief analysis shows that St costs about $0.06/kg, while HDPE costs about $0.86/kg. Replacing 40% HDPE would result in a 37% cost reduction. Each kilogram of St40 WPC produced would save $0.32, corresponding to $320/ton. Furthermore, carbon emissions would be reduced by substituting a nonrenewable material (HDPE) with natural St.

### 3.3 Physical Properties of the WPCs

Table 3 displays the density, moisture content, and water retention measurements. The density of the WPCs ranges from 780 to 987 kg/m^3^. The results indicate a linear increase with the rise in St levels in the WPCs. The composite rich in St exhibits the highest density. The 0 and 40 St WPCs show a difference of 207 density units. Cellulose is the most abundant component in wood fibers. The linear structure of cellulose facilitates self-assembly and the establishment of polymer entanglements. The high content of −OH sites in cellulose favors the interaction with FG. However, hydrophobic interactions in cellulose chains stem from its structural anisotropy and favor the interaction with HDPE. Cellulose chains can align in parallel, forming hydrophobic surfaces where the −OH is less exposed.^21^ Therefore, the presence of cellulose contributes to increasing the compatibility between FG and HDPE in the composite. The combination of these factors leads to the formation of compact and dense materials. Furthermore, density variations are related to the WPCs’ process and composition. The arrangement of precursors during the heating and cooling influences the physical properties of the material.^22^

**Table 3:** Physical Properties of the Composites

Composite	Density (kg/m^3^)	Moisture (%)	Water absorption (%)
HDPE	872	0.12^b^ ± 0.00	0.03^c^ ± 0.00
0 St	780	0.12^b^ ±0.00	0.83^b^ ± 0.0
10 St	810	0.86^a^ ± 0.08	2.40^a^ ± 0.09
20 St	840	0.82^a^ ± 0.05	2.38^a^ ± 0.08
30 St	910	0.89^a^ ± 0.05	2.37^a^ ± 0.06
40 St	987	0.87^a^ ± 0.01	2.45^a^ ± 0.06

HDPE-based composites reinforced with wood flour (40–60% w/w) exhibited densities ranging from 1047 to 1097 kg/m^3^.^5^ Philipp, Johannes, and Andreas produced WPCs composed of recycled/virgin HDPE enriched with wood particle residues obtained from postconsumer pallets (40–60% w/w). The authors reported results between 1050 and 1170 kg/m^3^.^23^ Both studies report increased WPC density with St added.

WPCs exhibit moisture contents below 1% w/w. The control material and 0 St show the lowest results. Composites enriched with St retain moisture seven times more. However, the measurements are equal in all composites St-loaded (*p* ≤ 0.05). WPCs show a similar behavior for water absorption. Only the St-discharged materials (HDPE and 0 St) show a statistical difference after 24 h of water immersion (*p* ≤ 0.05). The St comprises cellulose, a polysaccharide-rich in −OH groups.^23^ The −OH groups interact strongly through H–bonds with water molecules, justifying the findings in Table 3.^24^ WPCs composed of recycled and virgin HDPE containing 30 and 60% w/w of St showed water retention of 1.38 and 1.28% w/w, respectively.^23^

### 3.4 FTIR

**Figure 3:**
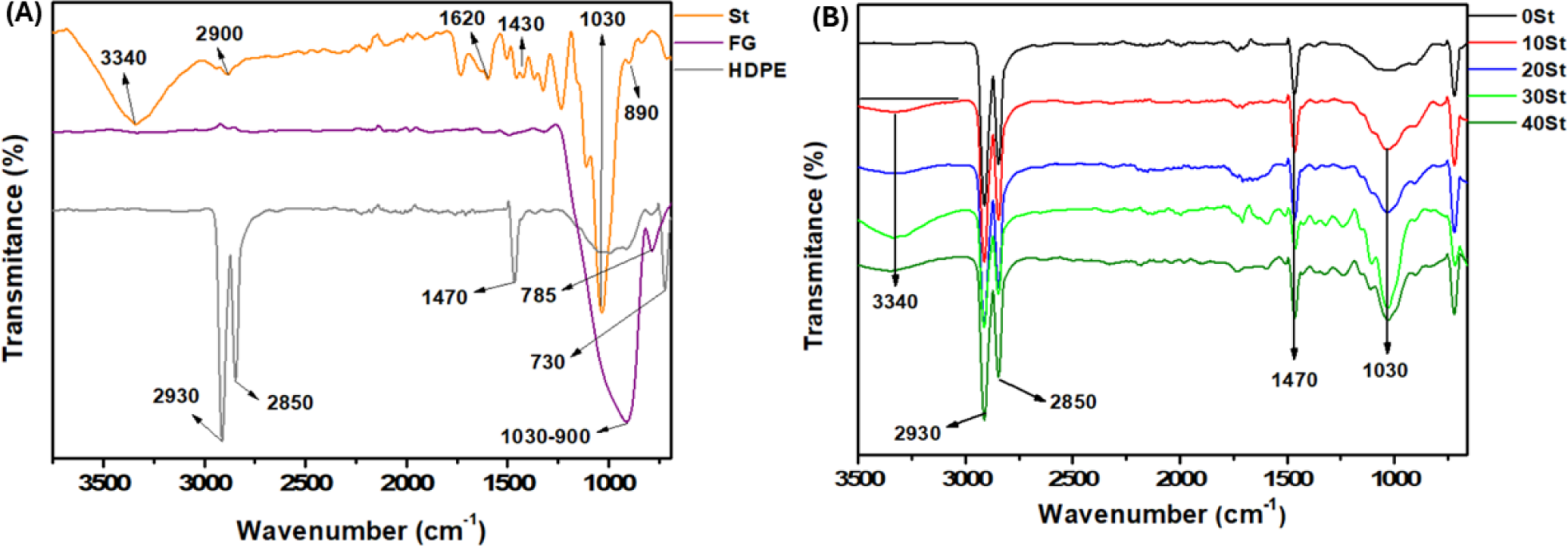
Spectra of the precursors (A) and composites (B).

The FTIR spectra for the precursors (St, FG, and HDPE) and composites (0, 10, 20, 30, and 40 St) are presented in Figure 3A,B. The HDPE spectrum shows peaks at 2800 and 3000 cm^–1^, attributed to C–H stretching vibrations. The signals identified at 1470 and 730 cm^–1^ correspond to in-plane and out-of-plane C–H bending vibrations, respectively.^25^

The St spectrum demonstrates a broad band at 3340 and 2900 cm^–1^, ascribed to the −OH and −CH groups of cellulose.^26^ Additionally, aromatic groups (lignin) and the crystalline and amorphous parts of cellulose at carbon six (C6) provide peaks at 1620, 1430, and 890 cm^–1^.^27^ The FG exhibits a band between 700 and 1300 cm^–1^, corresponding to the Si–O bonds.^28^ The precursors and composites exhibit the same signals. The main difference is attributed to a broad band at 3500–3000 cm^–1^. The signal corresponds to the – OH groups present in St, which are not identified in the 0 St spectrum. The absence of St in the 0 St composite justifies this. Similar spectra were reported by Morchid et al. in WPCs based on HDPE.^5^

### 3.5 DSC

**Figure 4:**
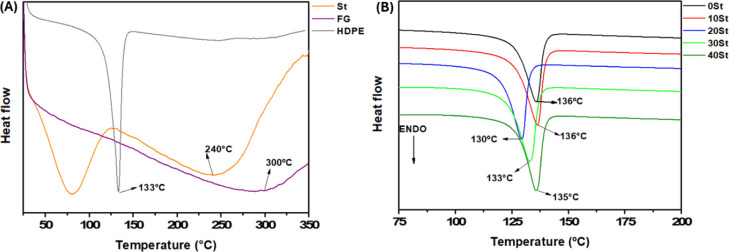
DSC curves of the precursors (A) and composites (B).

Figure 4 despite the DSC curves for the precursors and composites. Figure 4A suggests two endothermic peaks (80 and 240 °C) for St. These peaks correspond to water loss and the glycosidic bond rupture in polysaccharides.^29,30^ HDPE exhibits a melting peak at 133 °C.^31^ The FG showed an undefined endothermic signal near 300 °C. In contrast, the composites exhibit a curve like HDPE, containing an endothermic between 130 to 136 °C. The composition of the mixture justifies these measurements. The composites are based on 60% w/w HDPE and 40% w/w precursors. Thus, HDPE governs the interactions within the material. A previous study reported similar thermal features for WPC composed of HDPE reinforced with St (40–60% w/w) and compatibilized with maleic anhydride (2% w/w).^5^ Furthermore, the shifts in melting temperature due to the addition/removal of natural/synthetic fibers indicate changes in the intermolecular interactions within the WPCs. These findings support the formation of new composites.

### 3.6 SEM

**Figure 5:**
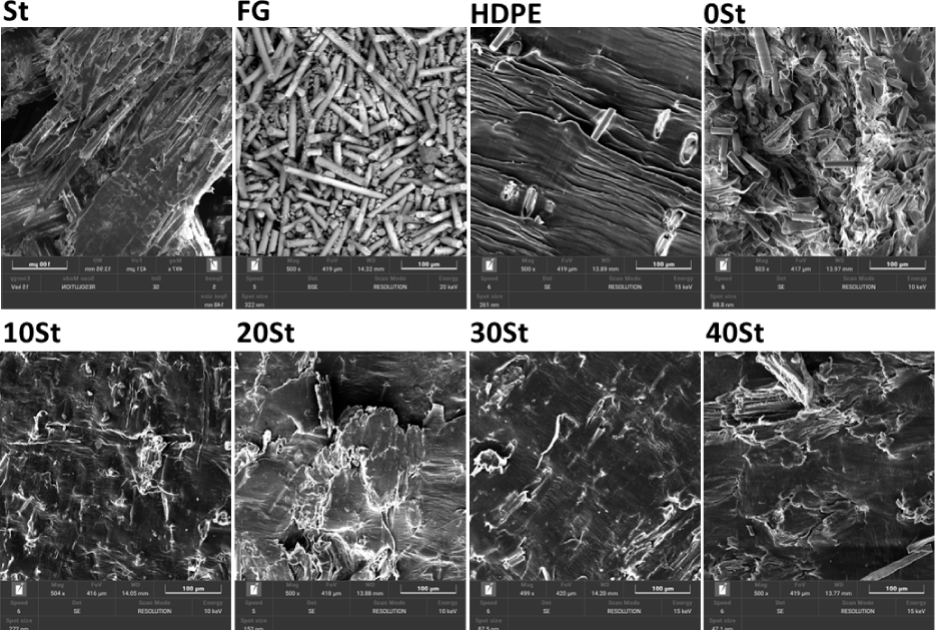
SEM images of the precursors and composites.

The micrographs of the WPCs and original materials are presented at 500× magnification (Figure 5). St is easily visualized in the SEM images, displaying a fibrous material with an irregular surface. FG exhibits a regular surface with cylindrical geometry of ranging sizes. HDPE shows a regular, slightly rough surface, suggesting a compact material.

In contrast, the micrographs of the composites show different characteristics. The images related to the 0 St material suggest a low interaction between the FG and the matrix. These findings are consistent with the digital images obtained in Figure 1, where multiple defects are observed in the material. The samples loaded with 10, 20, and 30% St exhibit the opposite behavior. There is no dispersed fiberglass on the composite surface, suggesting compatibility between St, FG, and HDPE, which also corroborate the digital images in Figure 1. Thus, St acts as a natural compatibilizer between HDPE and FG, enhancing the mechanical properties of the composites. Other studies have reported smooth surfaces in composites enriched with wood residues.^23,32,33^

### 3.7 EDS

**Figure 6:**
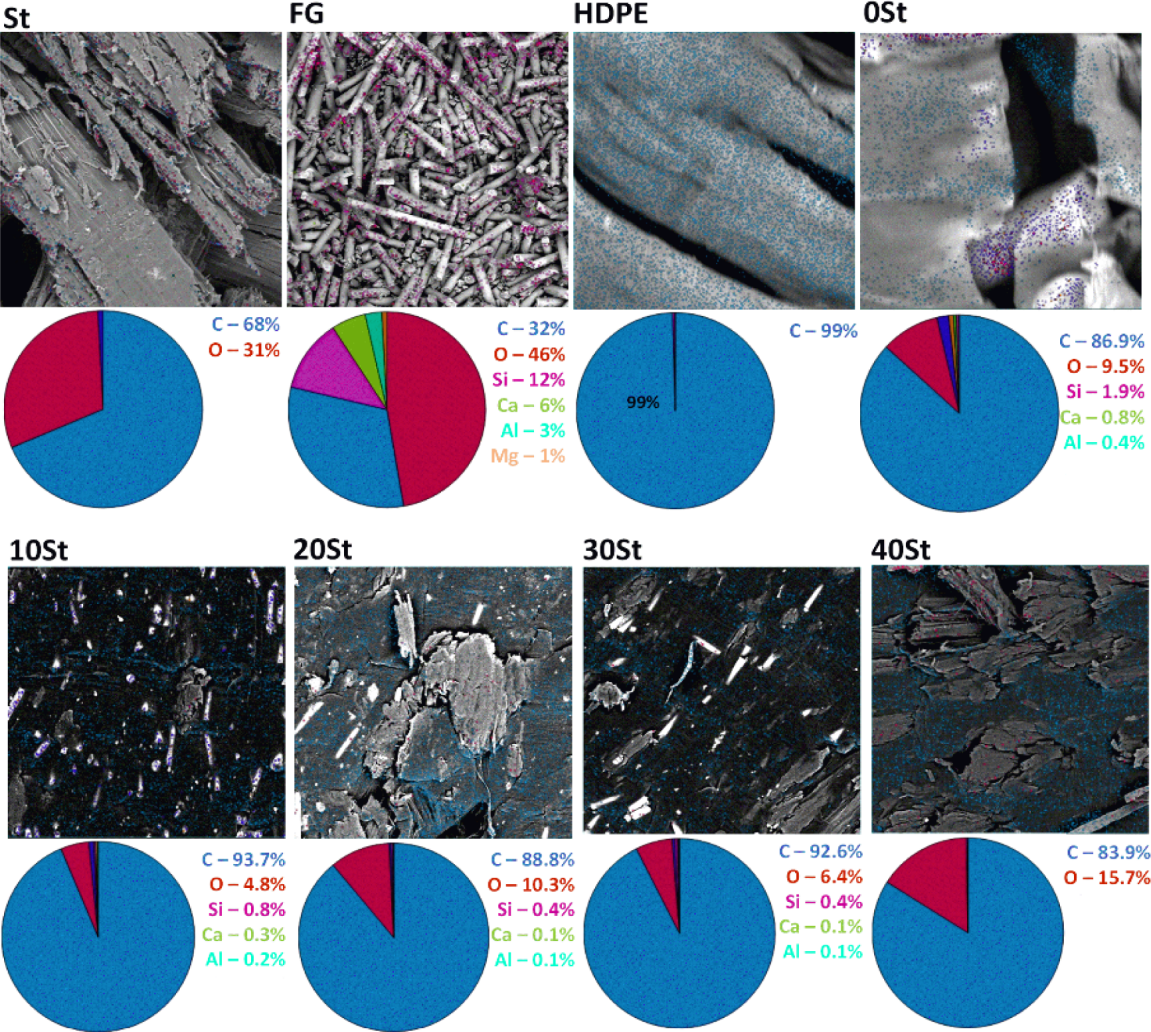
Mapping and elementary compositions of the precursors and composites.

The EDS mapping shows the atomic composition on the material surfaces (Figure 6). The St shows an atomic profile of 68% carbon and 31% oxygen. The wood fibers comprise natural macromolecules rich in carbon and oxygen, like cellulose, hemicelluloses, and lignin. The FG material exhibits a high carbon and oxygen content. Carbon is typically associated with resins and oxides with oxygen. Silicon, calcium, aluminum, and magnesium oxides are commonly employed in mixtures to enhance the composite mechanical properties. Only carbon was identified in the HDPE material.

The elemental atomic composition is the same across the composites except for silicon (absent in the 40 St). However, its relative element concentration depends on the composite’s mixture. These findings support the precursor transfer to the HDPE. Additionally, mapping atoms on the material’s surface provides insights into their distribution. The EDS spectra indicate the heterogeneity of the precursors in the 0 St composite. Silicon and carbon occur in different regions, a pattern not observed in 10, 20, 30, and 40 St WPCs where the atoms are homogeneously distributed. The EDS results agree with the SEM micrographs and digital images in previous sections. WPCs produced from mixed plastic waste (polyethylene, polypropylene, and polyethylene terephthalate) loaded with 70% w/w wood fibers were mapped by EDS. The spectra show the homogeneous distribution of the precursors, indicated by the random allocation of carbon and oxygen.^9^

### 3.8 Mechanical Properties

**Figure 7:**
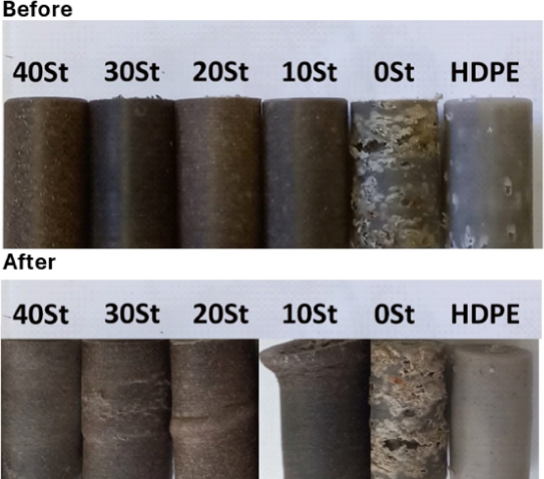
Digital images of the composites before and after the compression test.

Figure 7 shows the specimens before and after compression mechanical tests. The maximum values in kN for each composite were as follows: 10.89 (40 St), 7.90 (30 St), 5.63 (20 St), 5.7 (10 St), 0.97 (0 St), and 8.93 (HDPE) (Table 1). The 0 St composite exhibited the lowest compression resistance. These measurements are consistent with previous findings, particularly with the digital images, micrographs, and EDS spectra, which indicated surface flaws and precursor heterogeneity. Conversely, the composites containing St exhibited the highest compression resistance. The 40 St WPC showed a 17.3% higher resistance than the control material based on HDPE. The predominantly linear cellulose structure (main fiber component) facilitated the interfacial interaction with the HDPE matrix. Mihaela reported that an increase in organized phases within mixtures reduces the mobility of individual macromolecule chains, leading to increased material rigidity.^34^

### 3.9 Principal Component Analysis

**Figure 8:**
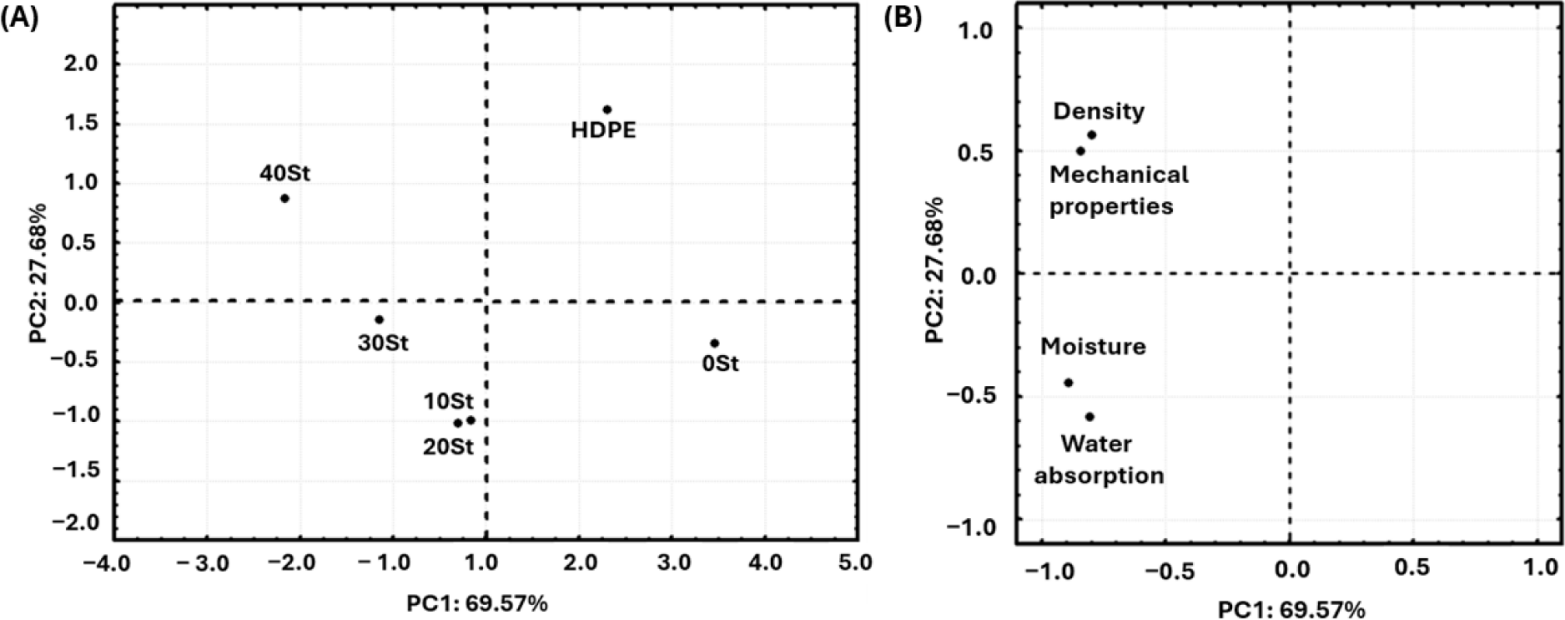
Scores plot (A) and loadings (B) of the composite characterization results.

A PCA evaluated the correlation between the composites (40, 30, 20, 10, and 0 St) and the control (HDPE) with the characterization results (water absorption (%), moisture content (%), density (kg/m^3^), and mechanical properties (kN)) (Figure 8). Two principal components (PCs) explain 97.25% of the total variance. PC1 accounts for 69.57%, while PC2 accounts for 27.69%. Ribeiro et al. also used two PCs to explain more than 90% of the data variance.^35^Figure 8A represents the projection of the principal components (PC1 (*x*-axis) and PC2 (*y*-axis)) against the composites. The characterization data influenced the formation of the groups. PC1 indicates three groups: the negative quadrant (40 and 30 St), near the center (20 and 10 St), and the positive quadrant (0 St and HDPE). PC2 also suggests the formation of a group in the positive quadrant (40 St and HDPE), near the center (30 and 0 St), and another in the negative quadrant (20 and 10 St).

On the other hand, Figure 8B indicates which measures support the formation of each group. For PC1, the proximity between the results of moisture content, density, water absorption, and mechanical properties justifies the allocation of the composites near the center (20 and 10 St), density and mechanical properties in the negative quadrant (40 and 30 St), and moisture content and water retention in the positive quadrant (0 St and HDPE). For PC2, density, and mechanical properties form the group of 40 St and HDPE in the positive quadrant; moisture content forms the group of 30 and 0 St near the center, and water absorption forms the group of 20 and 10 St in the negative quadrant. In conclusion, the map delineated by the PCs allows for identifying the best composition based on the desired property. For example, the 40 St composite was the most resistant to compression. The distance of 40 St from the other materials supports this. Thus, these results endorse the choice of the WPC 40 St, as indicated in the optimization.

## 4 Conclusions

The study shows WPCs produced from St/HDPE/FG mixtures. The optimization identified that a 40% w/w inclusion of sawdust resulted in the toughest WPC. Compression tests showed a 17.3% higher resistance increase than the control sample. The WPC demonstrates thermal stability and low moisture retention, making it suitable for applications in high-humidity and high-temperature environments such as beaches and rivers. However, its intermediate compressive strength qualifies it as a wood-like decorative material. Furthermore, substituting HDPE with an eco-friendly precursor reduces costs by about 37% and equivalent carbon emissions. Thus, 40 St WPC is economically advantageous and environmentally friendly.
